# Dataset of ultralow temperature refrigeration for COVID 19 vaccine distribution solution

**DOI:** 10.1038/s41597-022-01167-y

**Published:** 2022-03-02

**Authors:** Jian Sun, Mingkan Zhang, Anthony Gehl, Brian Fricke, Kashif Nawaz, Kyle Gluesenkamp, Bo Shen, Jeff Munk, Joe Hagerman, Melissa Lapsa, Nader Awwad, Chris Recipe, Doug Auyer, David Brisson

**Affiliations:** 1 Oak Ridge National Laboratory, One Bethel Valley Road, Oak Ridge, TN 37830 USA; 2 Carrier Global Corporation, 6304 Carrier Pkwy, East Syracuse, NY 13057 USA

**Keywords:** Mechanical engineering, Preventive medicine

## Abstract

Most COVID-19 vaccines require temperature control for transportation and storage. Two types of vaccine have been developed by manufacturers (Pfizer and Moderna). Both vaccines are based on mRNA and lipid nanoparticles requiring low temperature storage. The Pfizer vaccine requires ultra-low temperature storage (−80 °C to −60 °C), while the Moderna vaccine requires −30 °C storage. However, the last stage of distribution is quite challenging, especially for rural or suburban areas, where local towns, pharmacy chains and hospitals may not have the infrastructure required to store the vaccine at the required temperature. In addition, there is limited data available to address ancillary challenges of the distribution framework for both transportation and storage stages, including safety concerns due to human exposure to large amounts of CO_2_ from dry-ice sublimation, issues due to the pressure increase caused by dry-ice sublimation, and the potential issue caused by non-uniform cryogenic temperatures. As such, there is a need for test dataset to assist the development of a quick, effective, secure, and safe solution to mitigate the challenges faced by vaccine distribution logistics.

## Abstract (summary)

**Table:** 

Measurement(s)	temperature of air • weight of dry ice
Technology Type(s)	themocouple • scale
Sample Characteristic - Environment	climate system
Sample Characteristic - Location	united states

## Background & Summary

Along with development and manufacturing, vaccine distribution also presents great challenges for supply chain since most COVID-19 vaccines require ambient temperature control for transportation and storage. Two types of vaccine have been developed by manufacturers (Pfizer and Moderna). Both vaccines are based on mRNA and lipid nanoparticles requiring low temperature storage. The Pfizer vaccine requires ultra-low temperature storage (−80 °C to −60 °C), while the Moderna vaccine requires −30 °C storage^1^. Pfizer has designed a reusable package for transportation and storage that can keep the vaccine at the target temperature for 10 days and can accommodate between 1,000 and 5,000 doses^2^. However, the last stage of distribution is quite challenging^3^, especially for rural or suburban areas, where local towns, pharmacy chains and hospitals may not have the infrastructure required to store the vaccine at the required temperature^4^. Also, the need for a large amount of ultralow temperature refrigeration equipment in a short time period creates tremendous pressure on equipment suppliers^5^. In addition, there is limited data available to address ancillary challenges of the distribution framework for both transportation and storage stages, including safety concerns due to human exposure to large amounts of CO_2_ from dry-ice sublimation, issues due to the pressure increase caused by dry-ice sublimation, and the potential damage caused by cryogenic temperatures. As such, there is a need for a quick, effective, secure and safe product/solution to mitigate the challenges faced vaccine distribution logistics.

To address this gap, this study is to evaluate a local vaccine storage solution which uses refrigerated container technology to ensure a significant increase in vaccine viable time with reliable temperature control. Experimental data have been obtained to assess the technical merits of utilizing container refrigeration units with the ability to control temperature at −30 °C as part of the last mile supply chain for vaccine candidates, specifically,

- Investigate the temperature distribution of refrigeration storage container to assist thermal design optimization of cargo layout for the vaccine cartons for maintaining vaccine temperature within its required temperature tolerances.
- Study the CO_2_ concentrations inside the refrigeration storage container to support the determination of ventilation requirements and solution for maintaining acceptable CO_2_ concentrations inside the refrigeration storage container.

## Methods

The dataset was collected from two laboratory test platforms. A detailed description of test hardware and test platforms are given in the following sections.

### Refrigeration unit

The test unit is a refrigeration container unit of lightweight aluminum frame construction, designed to be bolted on to the front of a container and serve as the container’s front wall. The unit is self-contained and all electric unit, which includes cooling and heating systems to provide precise temperature control from −35 °C to 30 °C. The unit is supplied with a complete charge of refrigerant and compressor lubricating oil and is ready for operation upon installation. The base unit operates on nominal 380/460 volt, 3-phase, 50/60 hertz (Hz) power. An optional autotransformer may be fitted to allow operation on nominal 190/230 volt, 3-phase, 50/60 Hz power. Control system power is provided by a transformer which steps the supply power down to 12 and 24 volts, single phase. The unit uses a microprocessor controller which operates automatically to select cooling, holding or heating as required to maintain the desired set point temperature within very close limits. The controller has a keypad and display for viewing or changing operating parameters, such as various modes of operation.

### Vaccine packages

Two types of packages are used in this test: generic insulated box and pharmaceutical box. The specifications of these two packages are listed in Table 1,

**Table 1:** Specification of vaccine packages.

	Generic insulated box	Pharmaceutical box
Inside Dimensions (L × W × H)	16 3/4 × 16 3/4 × 15 (0.425 m × 0.425 m × 0.381 m)	15.16 × 11.22 × 9.84 (0.385 m × 0.285 m × 0.25 m)
Outside Dimensions (L × W × H)	20 3/4″ × 20 3/4″ × 19″ (0.527 m × 0.527 m × 0.483 m)	29.45″ × 25.51″ × 20.59″ (0.748 m × 0.648 m × 0.523 m)
Wall Thickness	2″ (0.051 m)	2.5″ (0.0635 m)
Internal payload volumes	68.8 L (0.069 m^3^)	28 L (0.027 m^3^)
Wall materials	Polystyrene foam	Polystyrene foam

### Test Instruments

The test units have been fully instrumented for monitoring the performance of proposed ultralow temperature refrigeration for COIVD 19 vaccine distribution solution, including temperatures, pressure difference, CO_2_/O_2_ concentrations. Specifically, type-T thermocouples are used to monitor the temperature profiles of refrigeration container, temperature of packages, supply and return air temperature of refrigeration system. CO_2_ and O_2_ concentration levels inside of refrigeration container unit are measured through Non-Dispersive InfraRed CO_2_ sensor and Figaro O_2_ sensor, both are pass through types. Omega Differential Pressure Transmitter measures the pressure difference between inside and outside of refrigeration container unit. Considering the low temperature working environment, mechanical scales are selected to weight dry ice during sublimation. More details of these instruments are given in technical validation section.

### Test platforms

**Fig. 1:**
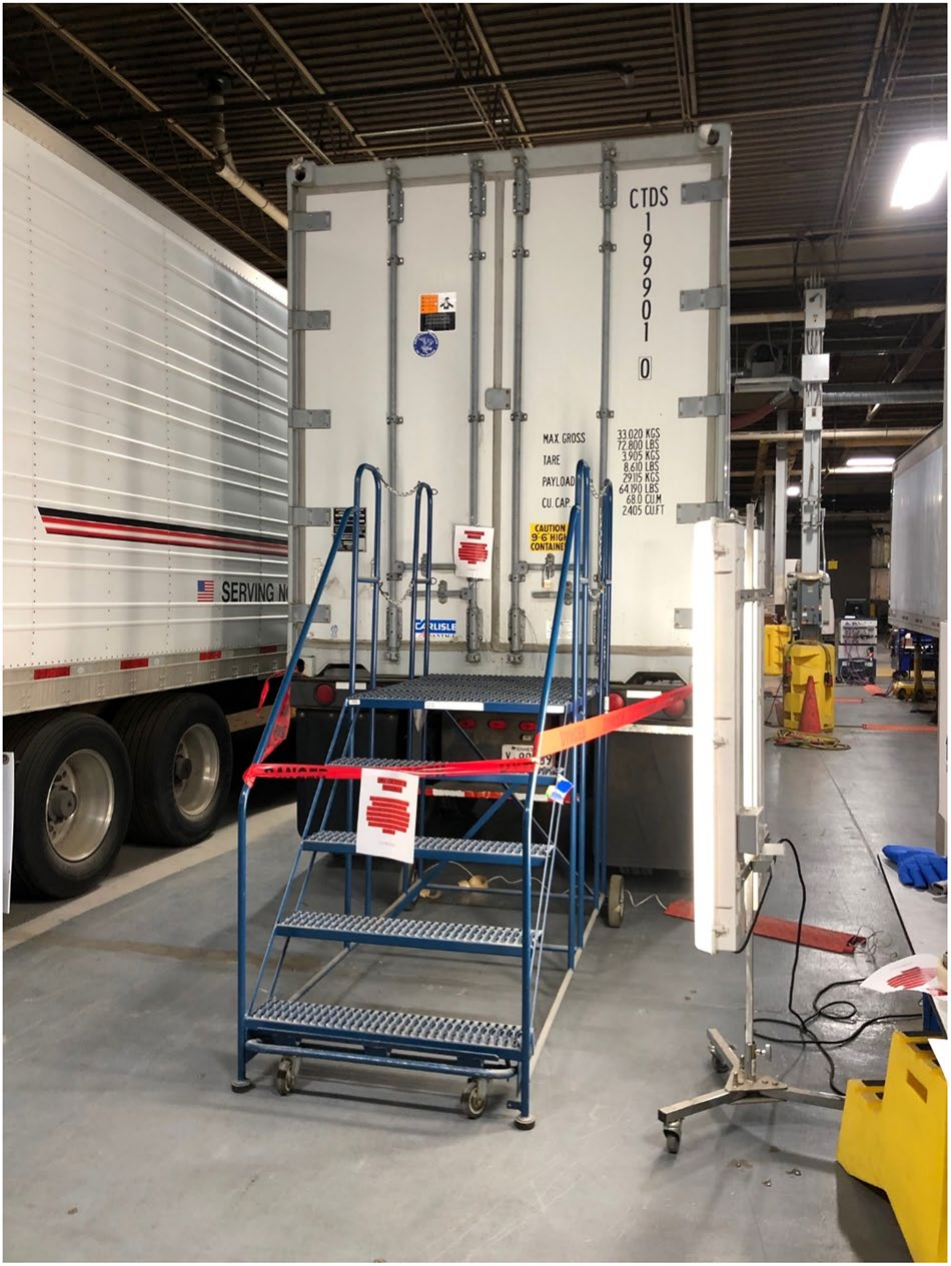
Test platform A at Syracuse NY.

Two test platforms have been established for this study. As shown in Fig. 1, the test platform A is set up at indoor lab space. A container refrigeration unit was used with attaching to a 40′ × 8′ × 8.6′ (12.2 m × 2.44 m × 2.62 m) / L × W × H refrigerated container.

The refrigeration storage container unit with attached container is fully instrumented with,

- Thermocouples, locations of thermocouples depend on different tests
- CO_2_ Sensor and O_2_ sensor to track the changes of CO_2_ and O_2_ concentration levels inside refrigeration container unit along with dry ice sublimation process.
- Pressure transducer to monitor the pressure differential from inside and outside the refrigeration container
- Mechanical scales to measure dry ice weight changes during sublimation to monitoring the dry ice sublimation process
- Third party real-time temperature monitors
- Third party temp monitoring system (AWS)

**Fig. 2:**
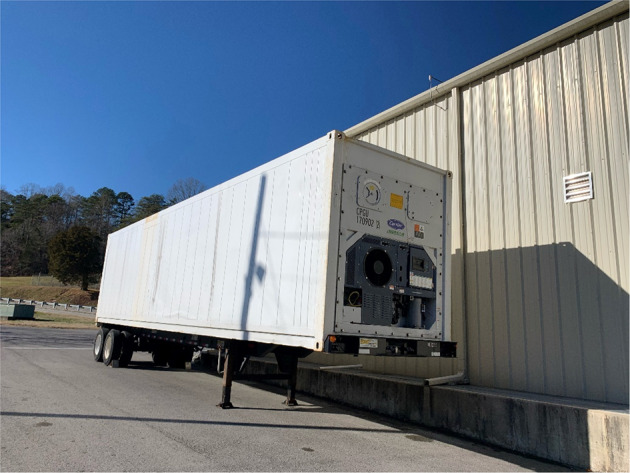
Test platform B at Oak Ridge TN.

For test platform B, the refrigeration storage container unit with attaching to a 40′ × 8′ × 8.6′ (12.2 m × 2.44 m × 2.62 m) / L × W × H refrigerated container is placed outside of building, as shown in Fig. 2.

The refrigeration storage container unit with attached container is fully instrumented with,

- Thermocouples, locations of thermocouples depend on different test.
- CO_2_ Sensor and O_2_ sensor inside container box, both near front at reefer.

The refrigeration system is energized with 460 V/60 Hz 3-phase power source from nearby building and controlled with manufacture provided controller, including data management service and air fresh vent function. The data acquisition system used in this test platform B is composed of Campbell Scientific real-time monitoring and control (RTMC) software, and CR 3000 Micrologger, a compact measurement and control datalogger housed in a portable, self-contained package, with RS-232 communication protocol.

## Data Records

The data were stored on figshare^6^, a shared platform, that can be accessed publicly. Table 2 summarizes the test data set, which comprises a collection of 2 comma-separated value (CSV) files.

**Table 2:** Files of the test dataset.

Data file	Size (M)	File description	Sample time
**Test 1: Test conducted in test platform A**			
Test1_TempCO2O2.csv	62.6	Temperature and CO_2_/O_2_ Concentration	10 sec.
Test1_DryIceWeight.csv	0.003	Weight of dry ice during sublimation	3 hours
**Test 2: Test conducted in test platform B**			
Test2_TempCO2O2.csv	7.3	Temperature and CO_2_/O_2_ Concentration	1 min.

The data includes two laboratory tests conducted in both test platform A and B. The test data record the air temperature distribution of refrigeration container unit, CO^2^and O^2^ concentration level of refrigeration container unit, and temperature inside of vaccine package. The details of each test are described as following,

### Test 1

**Fig. 3:**
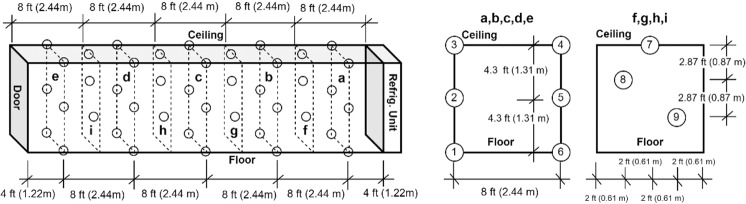
Location of thermocouples in refrigeration container in test platform A (Test 1).

**Fig. 4:**
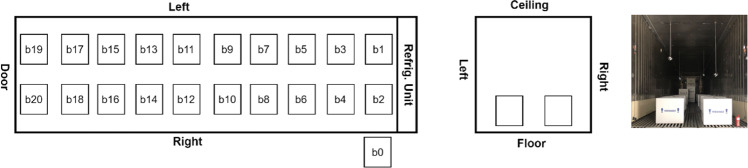
Location of dry ice packages in refrigeration container in test platform A (Test 1).

**Fig. 5:**
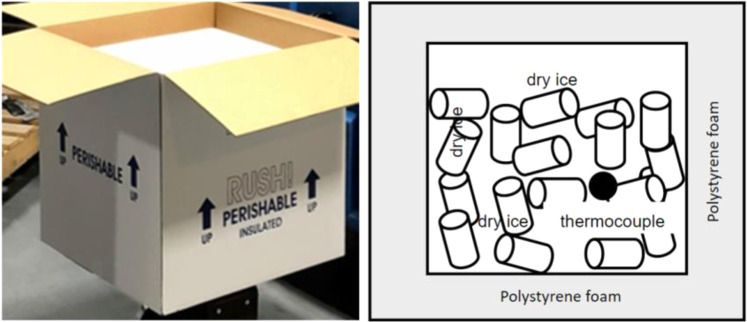
package with loaded dry ice (Test 1).

The first test was conducted in test platform A (Fig. 1). The thermocouples have been installed inside of the refrigeration container unit to monitor the temperature distribution. The locations of these thermocouples are shown in the Fig. 3. 20 Styrofoam boxes (b1 to b20) were placed inside of the refrigeration container unit in two rows along both side walls while one more box (b0) was placed outside in the ambient for comparison purposes. The placement of the boxes can be seen in Fig. 4. The dry ice is delivered and filled in these 21 boxes (b0 to b20) with approximate 50 lbs dry ice going into each of them. A thermocouple is installed inside each of these 21 boxes (Fig. 5), however the locations of thermocouples inside these boxes are not same for all boxes, which will lead the different characteristics of boxes temperature profiles in test 1.

To evaluate the dry ice sublimation, one of the boxes (b11 in the Fig. 4) on the inside of the container was placed on a mechanical scale with a closed caption television camera pointed at the scale. This was also repeated on the outside with the box b0 that was in the ambient for comparison purposes.

A set of O_2_ and CO_2_ sensors was utilized by using a diaphragm pump located at the rear of the unit. It was hooked up using plastic tubing to pump the air from the inside of the refrigeration container to the sensor through a hole going through the drain holes of the refrigeration container.

To monitor the pressure differential from inside and outside the refrigeration container, an Omega 5 V differential pressure monitor was installed at the rear of the unit with tube going through the refrigeration units frame and stretching to the pressure monitor.

Test starts with unit reach the setpoint temperature −34.5 °C and 100% open fresh air vent (FAV), and all temperatures are recorded with 10 seconds of sample time. Although CO2 was kept low concentration level, but it is unable to maintain the setpoint. FAV was closed after approximating 24 hours. Approximate 100 hours after the test staring, FAV was opened for dissipation of CO_2_ while still trying to maintain setpoint inside the refrigeration container. FAV was left open for remainder of test. The mechanical scale reading of boxes (b11 and b0) are recorded through closed caption television camera at 3-hour intervals.

### Test 2

**Fig. 6:**
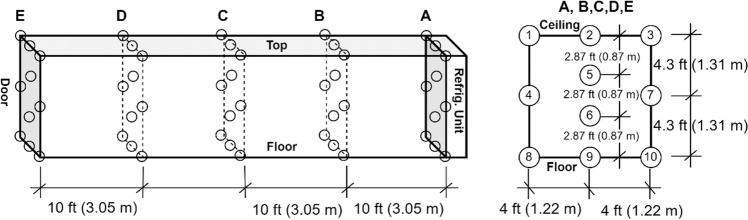
Location of thermocouples in refrigerated container in test platform B (Test 2).

**Fig. 7:**
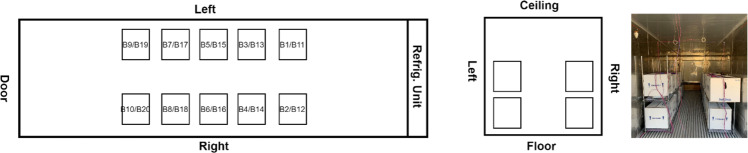
Location of thermocouples in refrigeration container in test platform A (Test 2).

**Fig. 8:**
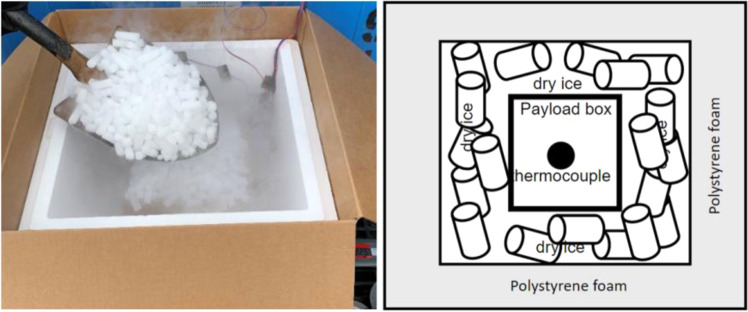
package with loaded dry ice and payload box insider (Test 2).

The second test was conducted in test platform B (Fig. 2). Thermocouples have been installed inside of refrigeration container unit to monitor the temperature distribution, as shown in Fig. 6. 20 Styrofoam boxes (B1 to B20) were placed inside of refrigeration container unit in two rows and two layers along both side walls while two boxes were placed inside of laboratory  for comparison purposes. The placement of the boxes can be seen in Fig. 7. An empty small payload box was set inside of each package with dry ice surrounding it, as shown in Fig. 8. A thermocouple was placed in the centre of each payload box to represent the vaccine temperature.

Similar to test 1, a set of O_2_ and CO_2_ sensors was utilized by using a diaphragm pump located at the rear of the unit. It was hooked up using plastic tubing to pump the air from the inside of the refrigeration container to the sensor through a hole going through the drain holes of the refrigeration container.

Test starts with unit reach the setpoint temperature −30 °C and closed FAV. FAV was left closed for entire test. All temperatures are recorded with 1 minute interval. Approximate 66 hours and 210 hours after the test staring, the back door of refrigeration container unit was open to investigate the changes of the CO_2_/O_2_ concentration levels inside refrigeration container unit during door-open period. The door was closed after the CO_2_/O_2_ concentration levels reaching safe values.

**Fig. 9:**
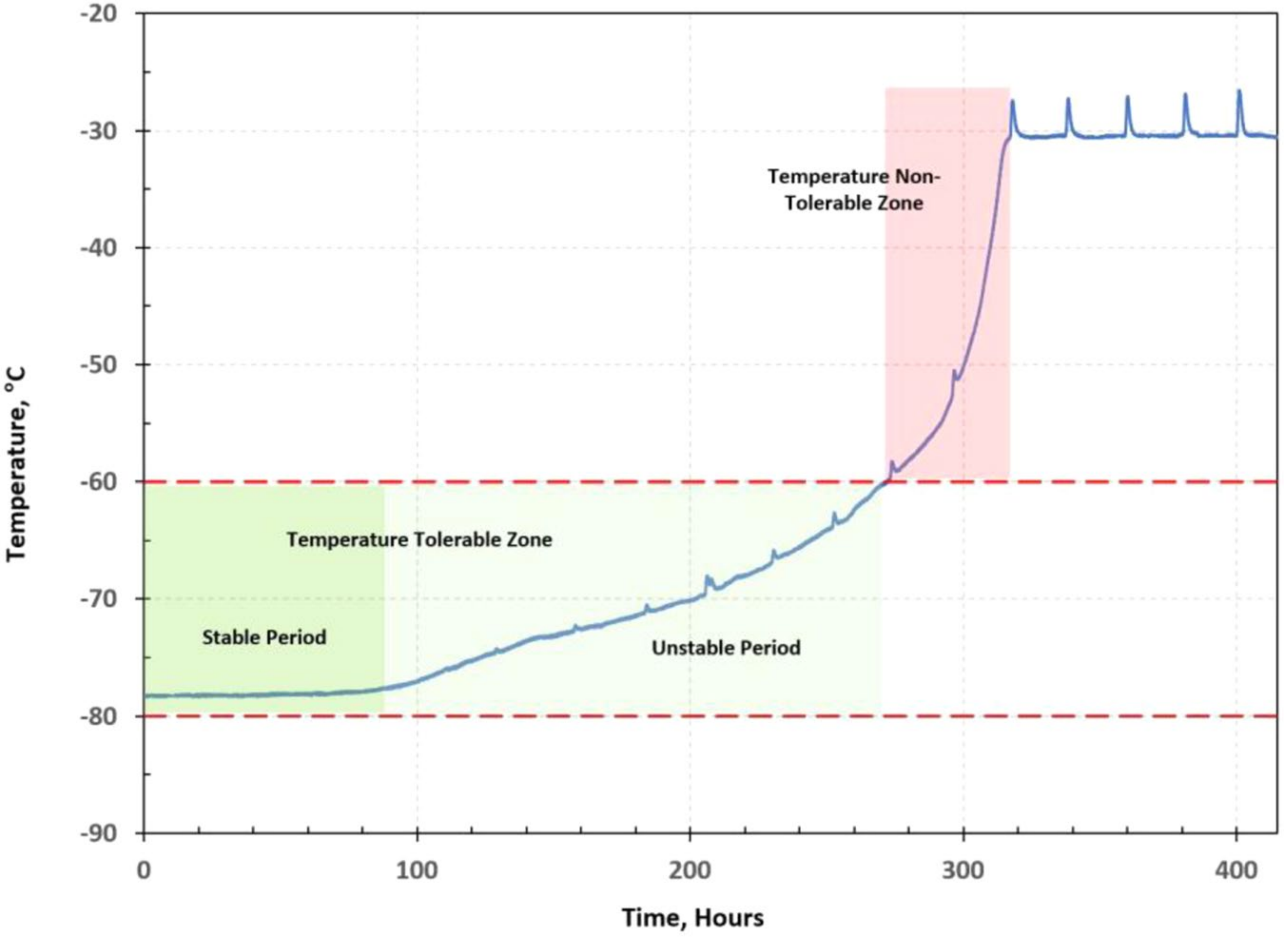
Generic temperature profile of vaccine payload box.

According to the test data, the temperatures of each payload box follow similar temperature profiles, a generic temperature profile of a vaccine payload box can be defined as shown in Fig. 9. The temperature profile can be divided into two regions: a temperature tolerable zone and a temperature non-tolerable zone. If the amount of dry ice left in the vaccine package can’t maintain the payload box temperature within the required temperature range of between −80 °C and −60 °C, it is in the temperature non-tolerable zone. Otherwise, it is in the temperature tolerable zone, which can be further divided into two stages: a stable period and an unstable period. In the stable period, the dry ice can keep the payload box at the dry-ice boiling point (sublimation point) temperature of −78.5 °C. When the payload box temperature starts to rise but is still within the required temperature range, it is in the unstable period of the temperature-tolerable zone.

## Technical Validation

The test refrigeration container unit and vaccine packages were fully instrumented to monitor the operational performance. The measurement includes temperature distribution inside refrigeration container unit, temperature inside vaccine packages, supply and return air temperature of refrigeration system, pressure difference between inside and outside of refrigeration container unit, CO_2_ and O_2_ concentration level inside refrigeration container unit. The detailed list of the measurement points and specification of the instrumentation are given in Table 3, and the technical quality of the data set can be understood through the accuracy of measurement.

**Table 3:** Specification of instruments.

Instruments	Measurement	Measurement Range	Accuracy
Type-T thermocouple	Temperature distribution of container and packages, Supply and return air temperature of refrigeration system	Element temp range is −195 °C(−320°F) to 371 °C(700 °F), insulation rated to 221 F	±0.5 °C (±0.9 °F)
CO_2_ sensor, NDIR Non-Dispersive InfraRed, pass through type, 5VDC input	CO_2_ level inside of container	1 to 4.7 VDC for 0 to 30% Vol. CO_2_ Concentration linear	±[0.2%vol. CO2 ± 3% of measured value] ± [20 mV ± 2% of measured volts]*8.11%/V
Figaro O_2_ sensor, Electrochemical Fuel Cell, pass through type, Amplified with 5 VDC input	O_2_ level inside of container	0.144 to Span Voltage (Calibration 20.8% Vol., value)	+/− 1%
Omega Differential Pressure Transmitter, Diaphragm Differential Pressure, 12 to 36 VDC input	pressure difference between inside and outside of refrigeration container unit	1 to 5 VDC for −10 to 10 IWG (Inch Water Gauge)	As good as calibrated 0.05%
Scale (Mechanical)	weight	0–31.75 kg (70 lbs)	0.567 kg (1/8 lbs)

### Simulation models

Beside the measurement, simulation models^7^ were also used to present the technical quality of the data set. A computational fluid dynamic (CFD) simulation model has been created using a commercial software, ANSYS/FLUENT (version 17.2)^8^, to replicate the experiments setup, including the size of the container and the size of the box, the distribution of the boxes. The sizes of the container and package (L × W × H) are 12.2 m × 2.4 m × 2.6 m and 0.53 m × 0.53 m × 0.48 m, respectively. A mesh of 1.7 million cells was generated for the simulation. Since the supply air is colder, the air inlets (supply air) locate at the floor of the container, where a series of T-shaped bars are used as diffuser to disperse the air flow. The return air locates at the top of the AC unite as the outlet of the flow loop of the container. The boundary conditions of the CFD model, including temperatures of the container walls, temperatures of the T-shaped bars, temperature of the supply air, and flow rate of the supply air are from the experiments. In this model, the adiabatic boundary conditions were applied to the boxes assuming that boxes are perfectly insulated. Equations of fluid flow and heat transfer are solved in the model. The buoyancy force effect is considered in the model by employing Boussinesq approximation due to the density change of air with various temperature. A standard *k-ε* model is used to describe the turbulence flow.

**Fig. 10:**
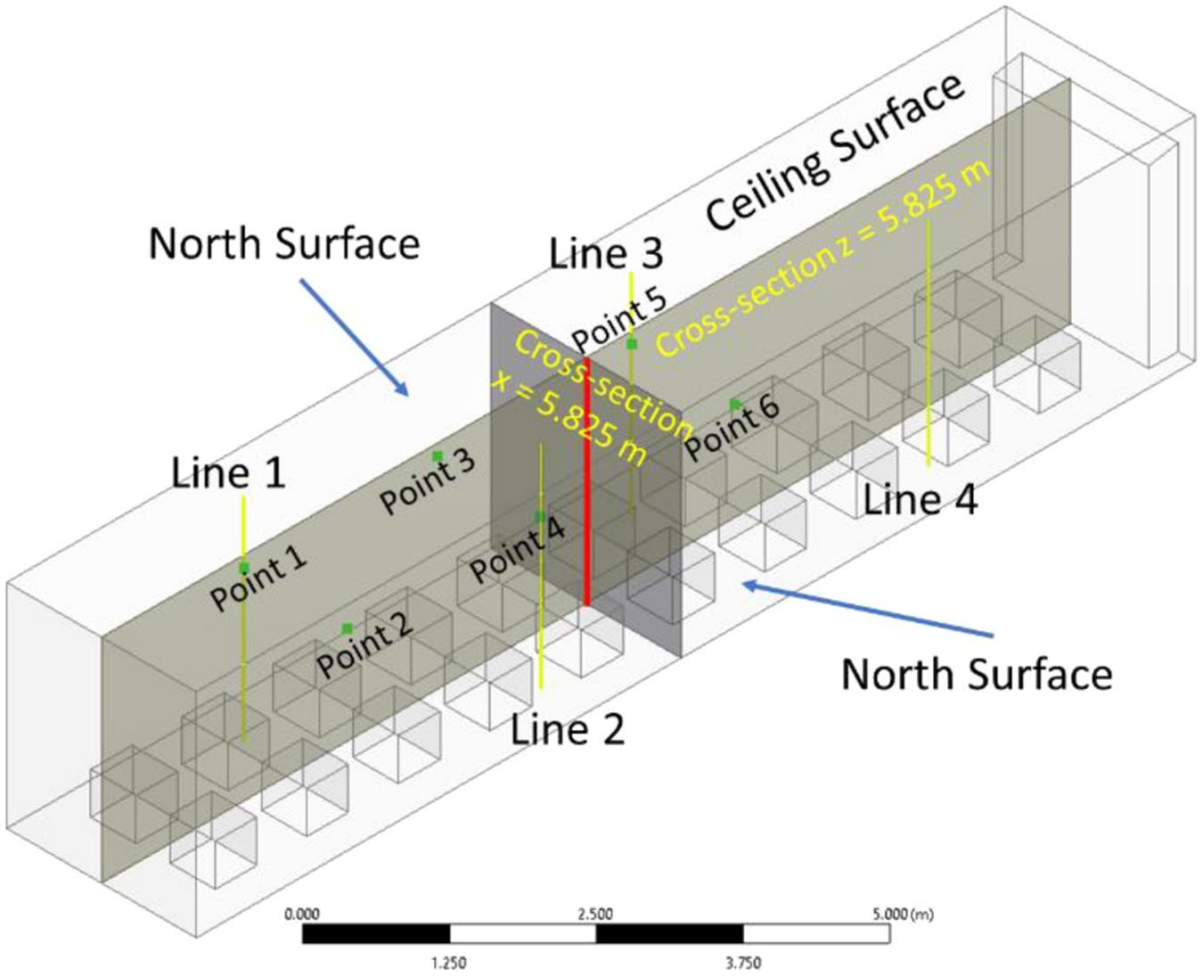
Locations of the six thermocouples to measure the temperature distribution in the container.

To validate the model, the experimental data are used to compared with steady-state simulation results generated by developed CFD model under the same conditions as the experiment. Temperatures were compared from six data points in the container (as shown in Fig. 10), as well as at the return air grille. Table 4 shows the comparison results. The temperatures from the CFD model were slightly lower than the experimental data, which may be due to the uncertainty in the testing. Nevertheless, the results show a reasonable agreement between experimental data and simulation results.

**Table 4:** The comparisons of temperatures from six data points and at the return air grille.

Data point	Model Prediction (°C)	Experiment (°C)
Point 1	−35.58	−34.5
Point 2	−35.40	−35.2
Point 3	−35.79	−35.5
Point 4	−35.80	−35.0
Point 5	−35.87	−34.8
Point 6	−35.59	−35.1
Return air	−35.73	−35.4

## Data Availability

No specific code was generated for analysis of these data.
